# The Glycaemic Index-Food-Frequency Questionnaire: Development and Validation of a Food Frequency Questionnaire Designed to Estimate the Dietary Intake of Glycaemic Index and Glycaemic Load: An Effort by the PREVIEW Consortium

**DOI:** 10.3390/nu11010013

**Published:** 2018-12-20

**Authors:** Elske M. Brouwer-Brolsma, Agnes A.M. Berendsen, Diewertje Sluik, Anne M. van de Wiel, Anne Raben, Jeanne H.M. de Vries, Jennie Brand-Miller, Edith J.M. Feskens

**Affiliations:** 1 Division of Human Nutrition and Health, Wageningen University, 6700 AA Wageningen, The Netherlands; agnes.berendsen@wur.nl (A.A.M.B.); diewertje.sluik@wur.nl (D.S.); anne.vandewiel@wur.nl (A.M.v.d.W.); jeanne.devries@wur.nl (J.H.M.d.V.); edith.feskens@wur.nl (E.J.M.F.); 2 Department of Nutrition, Exercise and Sports, Faculty of Science, University of Copenhagen, DK-1958 Copenhagen, Denmark; ara@nexs.ku.dk; 3 Charles Perkins Centre, School of Life and Environmental Sciences, University of Sydney, Sydney, NSW 2006, Australia; jennie.brandmiller@sydney.edu.au

**Keywords:** glycaemic index, glycaemic load, carbohydrates, FFQ, 24 h-recall, validation, dietary assessment

## Abstract

Dietary glycaemic index (GI) and glycaemic load (GL) are indices used to quantify the effect of carbohydrate quality and quantity on postprandial glycaemia. GI/GL-health associations are widely studied but data on the validity of integrated GI/GL measurements are scarce. We evaluated the performance of a food-frequency questionnaire (FFQ) specifically developed to assess GI/GL. In total, 263 Dutch men and 212 women (aged 55 ± 11 years) completed a 58-item GI-FFQ, an 183-item general-FFQ and a 2-day 24 h-recall and donated blood for glycated haemoglobin (HbA1c) determination. The level of agreement between these methods was evaluated by (1) cross-classification, (2) correlations and (3) Bland and Altman plots. The three dietary assessment methods provided comparable mean intake estimates for total carbohydrates (range: 214–237 g/day), mono/disaccharides (100–107 g/day), polysaccharides (114–132 g/day), as well as bread, breakfast cereals, potatoes, pasta, rice, fruit, dairy, cakes/cookies and sweets. Mean (±SD) GI estimates were also comparable between the GI-FFQ (54 ± 3), general-FFQ (53 ± 4) and 24 h-recalls (53 ± 5). Mean (±SD) GI-FFQ GL (117 ± 37) was slightly lower than the general-FFQ GL (126 ± 38) and 24 h-recalls GL (127 ± 37). Classification of GI in quartiles was identical for the GI-FFQ and general-FFQ for 43% of the population (*r* = 0.58) and with 24 h-recalls for 35% of the population (de-attenuated *r* = 0.64). For GL, this was 48% (*r* = 0.65) and 44% (de-attenuated *r* = 0.74). Correlations between GI and HbA1c were low (*r* = −0.09 for GI-FFQ, *r* = −0.04 for general-FFQ and *r* = 0.07 for 24 h-recalls). In conclusion, compared to a general-FFQ and 24 h-recalls, the GI-FFQ showed a moderate to good relative validity for carbohydrates, carbohydrate-rich foods and GI/GL. No metric predicted HbA1c.

## 1. Introduction

Glycaemic index (GI) and glycaemic load (GL) are measures generally used to estimate and rank the postprandial glycaemic response to carbohydrate-containing foods. More specifically, the GI is based on the blood glucose response following the consumption of a given food relative to the blood glucose response following the consumption of a reference product (e.g., glucose solution or white bread). Using the GI, the GL can be calculated by multiplying the food specific GI with the consumed quantity of that food.

Associations between GI and GL with disease risk have been widely studied [1,2,3,4,5,6,7,8]. While dietary GI and/or GL have been associated with the risk of type 2 diabetes in meta-analyses [2,8], other data have been inconclusive [1,2,3,4,5,6,7]. The inconsistencies in these results may be related to several factors, including population characteristics, dietary habits, as well as methodological factors. Of note, GI and GL are often estimated using food frequency questionnaires (FFQs) [1,2,3,4,5,6,7,8]. However, of the many studies evaluating GI/GL-health associations, only a handful have validated their GI/GL assessment method [9,10,11,12,13,14]. Therefore, FFQs principally designed to assess GI/GL [12] and additional validation studies [13] are warranted.

The identification of biological markers associated with dietary GI and GL may also aid future studies examining associations between dietary GI/GL and human health [15]. A review by Vega-Lopez and colleagues (2018) [16] presented evidence from observational studies suggesting that positive associations exist between dietary GI and/or GL and fasting glucose [17,18,19,20], an acute measure of glycaemic control [21], but these associations have also been contested [22,23,24,25]. Although less extensively examined, similar results exist for a longer-term marker of glycaemic control, HbA1c [21], showing significant associations [17,19,25] as well as neutral associations [20].

To contribute to this evolving research field, we developed an FFQ to assess dietary GI and GL (the GI-FFQ) according to a standardized approach [26]. To date, this approach has shown promise for assessing the intake of energy and various nutrients, including macronutrients, dietary fibre and several vitamins [27,28,29]. Subsequently, this GI-FFQ was evaluated against a commonly used general-FFQ [27,28,29] and against 2-day 24 h-recalls. As the consumption of high-GI/GL foods have been associated with different metrics of glycaemic control [17,18,19,20,25,30], we also examined the level of agreement between the dietary assessment methods and glycaemic control. Specifically, as the FFQs evaluate habitual diet (e.g., with the previous month serving as the reference) and HbA1c reflects the average glucose concentrations over the past 1–3 months [21], we also explored the potential association between dietary GI, GL and carbohydrate intake and HbA1c.

## 2. Materials and Methods

### 2.1. Participants

Between June 2011 and February 2013, 2048 Dutch men and women aged 20–70 years, were enrolled in the National Dietary Assessment Reference Database (NDARD) database, which was established to obtain detailed information on the level and variation in dietary intake as assessed by FFQs, repeated 24 h-recalls, as well as urinary/blood biomarkers [26]. The NDARD database was developed to facilitate the development of new high quality FFQs and validation of existing FFQs. Once enrolled, eligible participants were invited to the study centre and randomly assigned to either the ‘FFQ group’ (*n* = 959) or the ‘24 h-recall group’ (*n* = 1089). All participants, that is, participants in the ‘FFQ group’ and participants in the ‘24 h-recall group,’ were invited to complete a general 183-item FFQ. Given the unique nature of the NDARD database, the NDARD database was expanded by collecting extensive data on participant characteristics, including cross-sectional and longitudinal data on demographics, lifestyle, medical history and (cardio metabolic) health outcomes, called the Nutrition Questionnaires plus (NQplus) study [31]. Participants were recruited from the surroundings of Wageningen, the Netherlands and were able to speak and write Dutch. All participants gave written informed consent. The study was approved by the ethical committee of Wageningen University (approval code: NL34775.081.10) and was conducted according to the declaration of Helsinki. 

### 2.2. Population for Analyses

The current analyses were conducted using general-FFQ data (*n* = 1462), GI-FFQ data (*n* = 1064), 24 h-recall data (*n* = 1089) and data on glycated haemoglobin (HbA1c) (*n* = 1871). Merging the different subsets resulted in a sample of *n* = 493 with complete data on all four subsets. Participants with potentially unreliable or incomplete general-FFQ, GI-FFQ and 24 h-recall data [i.e., men with energy intakes <800 kcal or >4200 kcal, women <500 kcal or >3500 kcal (*n* = 18)] were excluded [32]. In total, data on 475 participants were available for analyses, which included data of 263 men and 212 women, aged (mean ± SD) 55 ± 11 years, with a body mass index (BMI) of 25.5 ± 3.7 kg/m^2^. Sixty-six percent of the participants completed higher education, 52% never smoked and 7% indicated that they followed a diet.

### 2.3. Glycaemic Index FFQ

We developed a new (short) FFQ to assess GI and GL. Data from the Dutch national food consumption survey 2007–2010 (*n* = 3819) was used to select the appropriate food items for the FFQ. Two main factors were calculated, including (1) the relative contribution of the food items to dietary GL and (2) the explained variance (R^2^) in dietary GI and GL with stepwise linear regression. The first step was applied to select foods with the highest GL or foods eaten in very large amounts, contributing to 80% of the total GL, irrespective of GI, including breads (all types), potatoes, dough used in pizza and savoury pies, chips, pasta, rice, cake, sugar-sweetened beverages (soft drinks, juice, fruit drink, ice tea), milk, beer and fruit (banana, apple). The second step was applied to select the food items explaining the largest between-person variation in dietary GI and GL. Together these two steps resulted in a selection of food items that contributed most to the total GL and explained most of the variance in GL. Forty-seven food items explained 90% of the variance in GL. For GI, the maximum amount of variance that could be explained was 48% with 123 food items. Foods explaining the highest amount of variance in GI included bread and rolls (all types), French fries, rice, potatoes, sugar-sweetened beverages, sweets and beer. To note, low-GI foods (red wine, milk and peanuts) also scored relatively high. Foods accounting for the highest variance in GL were rather similar, including bread, rice, pasta, potatoes, sugar-sweetened beverages and beer. Moreover, to optimize the food-item selection even further, two additional analyses were performed. First, stepwise logistic regression was performed on all individuals from the first quartile (Q1; case = 0) and the fourth quartile (Q4; case = 1) of dietary GI and GL, including all food items as independent variables. For GI, the odds ratios were significantly positive for bread and rolls (all types), biscuits, sponge cake, beer, soft drinks, fruit drinks, juices, sweets, pancakes, rice, pasta, potatoes, chips and tomatoes. The odds ratios were significantly negative for sweetcorn, milk chocolate, milk, custard, yogurt drink, juice drinks, apple juice, peanuts, bacon, salami and zucchini. Stepwise logistic regression for dietary GL only selected food items that had positive significant odds: breads, pasta, rice, yogurt drinks, soft drinks, fruit drinks and juices, beer, sugar and sweets, jams, biscuits, chocolate milk and chocolate spread. Based on these results, we decided to additionally assess the intake of vegetables, peanuts and peanut butter and chocolate (spread). Second, a principal component analysis was conducted within Q1 and Q4 of dietary GI and GL, yielding three factors. The food groups with factor loadings >0.20 or ≤0.20 for each of the three factors after rotation were included in the pattern. In the lowest GI-quartile, the first factor was characterized by a high intake of vegetables, cheese, beef, fish, vegetable oil, coffee, tea, water and wine and a low intake of milk beverages, salty biscuits, deep frying fats, chocolate, confectionary, biscuits, soft drinks and snacks. In the highest GI-quartile, the first factor was characterized by a high intake of vegetables, pasta and rice, chicken, vegetable oil, coffee and sauces. However, as these factors included many foods with no carbohydrates, we decided not to include additional food items based on this dietary pattern analysis. The combination of these approaches eventually resulted in 58 FFQ-items (Table S1). GI was calculated through multiplying the consumption frequency by portion size and GI as indicated in the GI database prepared by Sluik and colleagues [33]. Dietary GI and GL were derived by the weighted mean of GI and GL of all food items included in the FFQ by means of commonly used formulas [34]. The GI-FFQ used in this study is available on request (in Dutch).

### 2.4. Food-Frequency Questionnaire

Habitual dietary intake was assessed by a semi-quantitative FFQ including 183 items, which has been validated for intakes of energy, macronutrients, dietary fibre and a selected number of vitamins [27,28,29]. This FFQ covered ≥96% of the absolute level of intake and ≥95% of the between-person variability of each nutrient as assessed in the 1998 Dutch national food consumption survey [35]. Questions relating to consumption frequency were followed by answer categories ranging from ‘never’ to ‘6–7 days per week.’ Portion sizes were estimated using natural portion sizes and commonly used household measures. Subsequently, nutrient intakes were calculated through multiplying the consumption frequency by portion size and nutrient content (grams) as indicated in the Dutch food composition table [36]. GI and GL were calculated by the same approach as for the GI-FFQ.

### 2.5. Phone-Based 24 h-Recalls

Participants completed two phone-based 24 h-recalls. Dates for telephone-based 24 h-recalls were randomly selected. When recall attempts were denied, the recall was randomly rescheduled within 3–10 days. Recalls were performed by experienced dieticians according to a standardized protocol and using the five-step multiple pass method [37,38,39,40]. Portion sizes were assessed using commonly used household measures, weight/volume and standard portions. The recalls were transcribed into the food codes of the 2011 Dutch food composition table [35]. GI and GL were calculated by the same approach as for the GI-FFQ. Regular meetings with all dieticians and quality checks ensured quality of the telephone calls and encoding of the data. Furthermore, information on supplement intake and whether a diet regime was followed during the past month, prescribed or at own initiative, were registered. A more detailed protocol of the telephone-based 24 h-recalls can be found in the study design paper of the NDARD-project [26].

### 2.6. Blood Sampling

Fasting blood samples were collected in the morning at hospital Gelderse Vallei (Ede) or hospital Rijnstate (Velp). Samples were analysed in the respective hospital laboratories and used the same methodology and standardized protocols for risk factor assessments. Both had external quality control in the Netherlands (SKML). HbA1c analyses were performed using Dimension Vista 1500 automated analyser (Siemens, Erlangen, Germany) or Roche Modular P800 chemistry analyser (Roche Diagnostics, Indianapolis, IN, USA).

### 2.7. Additional Measurements

Participants completed the health and lifestyle questionnaires online, using the open-source survey tool LimesurveyTM (LimeSurvey Project Team/Carsten Schmitz, Hamburg, Germany). The questionnaires included questions on demographics, educational attainment and smoking habits, amongst other questions. These general questions were predominantly derived from questionnaires used in the Lifelines study [41]. Physical examinations were conducted by well-trained staff at the study centre according to a standardized protocol. Height was measured with a stadiometer (SECA, Hamburg, Germany) to the nearest 0.1 centimetre, without shoes. Weight was measured on a digital scale (SECA) to the nearest 0.1 kg, without shoes and sweaters and with empty pockets. No corrections were made for other clothes such as jeans, skirts, sweatpants or t-shirts. BMI was calculated as weight/height^2^.

### 2.8. Statistical Analysis

Participant characteristics are reported as mean with standard deviation (mean ± SD), or *n* with percentages (*n*, (%)). Means with SD are also provided for intakes of energy, carbohydrates, food groups and GI and GL estimates. We examined both absolute as well as energy-adjusted nutrient and food intake, where energy-adjustment was applied using the commonly used residual method [42]. To assess the ability of the GI-FFQ to rank individuals according to their intakes of nutrients, foods, GI and GL, intakes obtained by GI-FFQ, general-FFQ and 2-day 24 h-recalls, as well as HbA1c concentrations, were divided in quartiles (or tertiles as specified below the tables). We then examined whether individuals were ranked into the same, adjacent or nonadjacent quartile. If ≥50% of the participants were classified in the same tertile or quartile this was considered a good outcome [43]. Thereafter, Spearman correlation coefficients between GI-FFQ and general-FFQ and the mean of 2-day 24 h-recalls were calculated; correlations are shown as crude, energy-adjusted and de-attenuated (i.e., correlation divided by √average measure of the intra class correlation coefficient (ICC)), as well as energy-adjusted and de-attenuated. Moreover, Spearman correlations between GI-FFQ, general-FFQ and 24 h-recalls with HbA1c concentrations were also obtained. Correlations coefficients ≥0.50 were classified as good, 0.20–0.49 as acceptable and <0.20 as poor [43]. Finally, Bland-Altman plots were created in order to further examine the level of agreement between the dietary assessment methods, where the difference in the estimated mean GI and GL of the GI-FFQ with the general-FFQ and 24 h-recalls was plotted against the mean GI and GL as estimated by the dietary assessment methods. All analyses were conducted using the statistical package SPSS 22.0. A *p*-value ≤ 0.05 was considered statistically significant.

## 3. Results

Population characteristics are shown in Table 1.

The mean daily energy intake as estimated by the general-FFQ was 9054 ± 2113 kJ (conversion unit for kcal; kJ/4.2), which was comparable to the energy intake estimated by the 2-day 24 h-recalls (9084 ± 2028 kJ/day) (Table 2). As expected, the energy intake estimated by the GI-FFQ was substantially lower, at 5854 ± 1725 kJ/day. All three methods provided comparable estimates for total carbohydrates (range 214–237 g/day), mono- and disaccharides (range 100–107 g/day) and polysaccharides (range 114–132 g/day). Absolute intakes of most carbohydrate-rich food groups (e.g., bread, breakfast cereals, potatoes, pasta, rice, fruit, dairy, cakes and cookies and sweets) were rather similar; as indicated by the SDs, the data of the 2-day 24 h-recalls showed the largest variation for most food groups. Absolute intake estimates for vegetables and alcoholic beverages were substantially lower according to the GI-FFQ as compared to the general-FFQ and 2-day 24 h-recalls. Absolute mean ± SD GI estimates were highly comparable for the GI-FFQ, general-FFQ and 2-day 24 h-recalls, namely 54 ± 3, 53 ± 4 and 53 ± 5, respectively. The GL measured by the GI-FFQ (117 ± 37) was slightly lower than the GL measured by the general-FFQ (126 ± 38) and 2-day 24 h-recalls (127 ± 37).

When categorizing the intake of energy and carbohydrates in quartiles, the GI-FFQ classified 45–48% of the participants in the same quartile as compared to the general-FFQ (Table 3). Stratified analyses for sex showed a better cross-classification for the intake of polysaccharides in women (52%, *r* = 0.59) than in men (45%, *r* = 0.57); however, no substantial differences were observed for energy, total carbohydrates, mono/disaccharides or fibres (Table S2). Correlation coefficients for energy and carbohydrates were good, ranging from *r* = 0.58 for energy to up to *r* = 0.64 for total carbohydrates. Compared to the 2-day 24 h-recalls, 39% (energy) up to 44% (polysaccharides) of the participants were classified in the same quartile as estimated by the GI-FFQ, where de-attenuated correlation coefficients were good, ranging from *r* = 0.60 for energy to up to *r* = 0.75 for polysaccharides.

Good cross-classification (≥50% in the same quartile or tertile) results for the GI-FFQ with the general-FFQ were observed for bread (54%), breakfast cereals (62%), fruit (52%), sugar-sweetened beverages (58%), fruit juices (51%) and alcoholic beverages (53%). Good correlations were observed for coffee (*r* = 0.79) and bread (*r* = 0.71), while correlations for fruit (*r* = 0.67), alcoholic beverages (*r* = 0.67), cakes and cookies (*r* = 0.62), fruit juices (*r* = 0.59), dairy (*r* = 0.58), pasta (*r* = 0.56), potatoes (*r* = 0.55) and sugar-sweetened beverages (*r* = 0.50) were moderate. Comparisons between the GI-FFQ with the 2-day 24 h-recalls showed good cross-classification (≥50%) for breakfast cereals (62%), coffee (55%) and sugar-sweetened beverages (51%). Good de-attenuated correlations were shown for coffee (*r* = 0.84), cake and cookies (*r* = 0.77), soup (*r* = 0.76), bread (*r* = 0.75), dairy (*r* = 0.71), fruit (*r* = 0.60), alcoholic beverages (*r* = 0.59), fruit juices (*r* = 0.56), sweets (*r* = 0.53), breakfast cereals (*r* = 0.51) and potatoes (*r* = 0.50).

**Figure 1:**
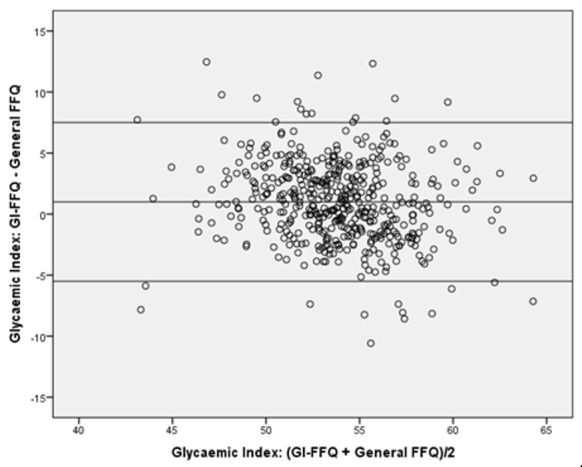
Bland-Altman plot for glycaemic index showing the relative validity of the GI-FFQ versus the general-FFQ. Middle line indicates the mean; upper and lower lines indicate borders based on mean ± SD × 1.96 (i.e., 1.0+/−6.5).

**Figure 2:**
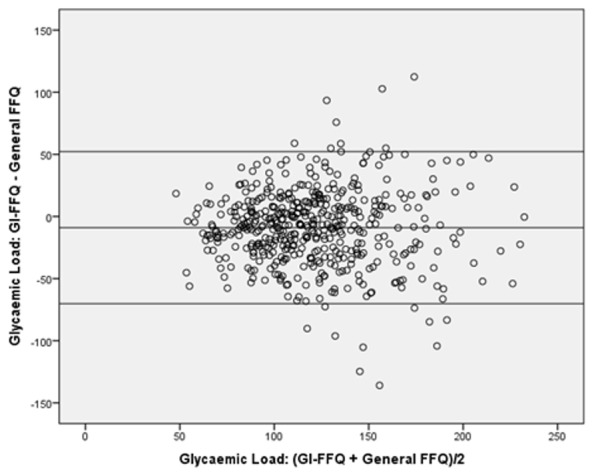
Bland-Altman plot for glycaemic load showing the relative validity of the GI-FFQ versus the general-FFQ. Middle line indicates the mean; upper and lower lines indicate borders based on mean ± SD × 1.96 (i.e., −9.0+/−61.2).

**Figure 3:**
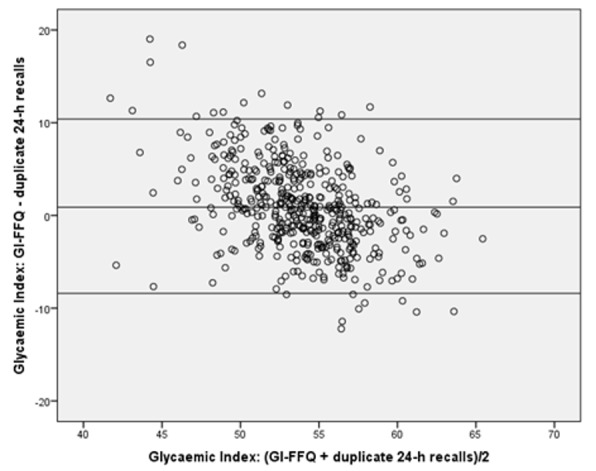
Bland-Altman plot for glycaemic index showing the relative validity of the GI-FFQ versus the duplicate 24 h-recall. Middle line indicates the mean; upper and lower lines indicate borders based on mean ± SD × 1.96 (i.e., 0.9+/−9.4).

**Figure 4:**
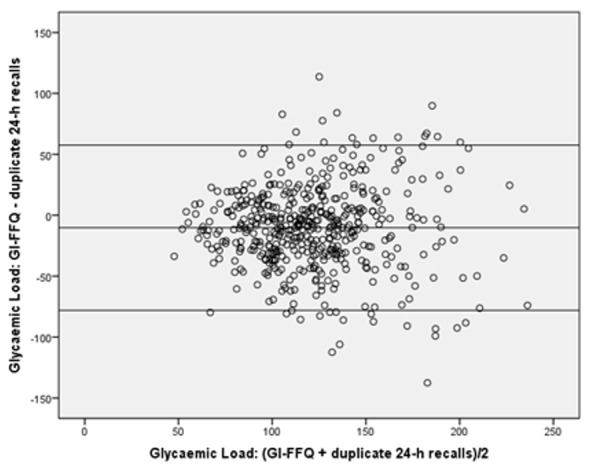
Bland-Altman plot for glycaemic load showing the relative validity of the GI-FFQ versus the duplicate 24 h-recall. Middle line indicates the mean; upper and lower lines indicate borders based on mean ± SD × 1.96 (i.e., −10.2+/−67.7).

For GI, the GI-FFQ classified 43% (*r* = 0.58) and 35% (de-attenuated *r* = 0.64) of the participants in the same quartile as the general-FFQ and 2-day 24 h-recalls, respectively; which was 48% (*r* = 0.65) and 44% (de-attenuated *r* = 0.74) when comparing GL estimates. Cross-classification did not point towards sex-specific differences for GI. However, for GL, the cross-classification results were better in men (45%, *r* = 0.58) than in women (41%, *r* = 0.62). Bland-Altman plots (Figure 1, Figure 2, Figure 3 and Figure 4) furthermore indicated a fair agreement of the GI-FFQ with the general-FFQ and the 2-day 24 h-recalls.

Correlations between the dietary GI, GL and carbohydrate measures with HbA1c were poor, ranging from *r* = −0.09 (with 23% classified in same quartile) for GI as assessed by the GI-FFQ, to up to de-attenuated *r* = 0.07 (with 26% classified in same quartile) for GI as assessed by the 2-day 24 h-recalls (Table 4).

## 4. Discussion

The GI-FFQ evaluated in this study was specifically developed to assess dietary GI and GL in the Dutch population for use in future epidemiological studies. As such, the GI-FFQ was developed to rank participants according to their dietary GI and GL intakes and not to obtain precise absolute intake estimates. Statistical analyses indicated a moderate to good relative validity for carbohydrates, carbohydrate-rich foods, GI and GL compared to an extensive general-FFQ and 2-day 24 h-recalls. Correlations between HbA1c with the GI, GL and carbohydrate intakes estimated by the GI-FFQ and the other dietary assessment methods were poor. Although correlations and cross-classification suggested a moderate to good relative validity for various other nutrients and food groups, the GI-FFQ was not developed to assess the overall intake of nutrients or food groups, which is clearly illustrated by the relatively low total energy intakes.

In our study, the GI-FFQ classified 43% (*r* = 0.58) and 35% (de-attenuated *r* = 0.64) of the participants in the same quartile as the general-FFQ and 2-day 24 h-recalls, respectively, when comparing GI estimates and 48% (*r* = 0.65) and 44% (de-attenuated *r* = 0.74), respectively, when comparing GL estimates. These cross-classification percentages and moderate to strong correlations are in line with results of other GI/GL-validations studies. A study conducted in a population of 141 Swedish men aged 40–74 years showed de-attenuated Pearson correlations between the average of two 96-item FFQs and two 1-week diet records of *r* = 0.62 for GI and *r* = 0.77 for GL [12]. The FFQs and diet records categorised 70% (GI) and 79% (GL) of the population in the same or adjacent quintile [12]. In the Blue Mountains Eye Study (BMES) comprising 78 men and women aged ~70 years, de-attenuated Spearman correlations of *r* = 0.57 (40% same quintile) for GI and *r* = 0.38 (31% same quintile) were observed for GL using three 4-day weighted food records and 145-item FFQ [9]. A Japanese study comprising 92 men and 92 women aged 31–76 years revealed de-attenuated energy-adjusted Pearson correlations of *r* = 0.72 (47% same quintile) (women) and *r* = 0.65 (38% same quintile) (men) for GI and *r* = 0.66 (41% same quintile) (women) and *r* = 0.71 (49% same quintile) (men) for GL using a 4-day 118-item FFQ and 4-day dietary records [13]. In the European Prospective Investigation into Cancer and Nutrition study, moderate (*r* = 0.57) and good (*r* = 0.76) ecological correlations were revealed between FFQ and a single 24 h-recall for GL and GI, respectively [14]. Pearson correlation coefficients were *r* = 0.63 for both GI and GL among 121 Dutch men and women aged 23–72 years completing an 178-item FFQ and twelve 24 h-recalls [10]. Additionally, in a Finnish study including data on 218 men and 292 women aged 25–74 years, comparisons between a 131-item FFQ and two 3-day food records showed energy-adjusted Spearman’s correlations for GI of *r* = 0.31 (65% same or adjacent quintile) and *r* = 0.43 (69% same or adjacent quintile) in men and *r* = 0.41 (69% same or adjacent quintile) and *r* = 0.49 (72% same or adjacent quintile) for GL in women [11].

As GL is related to the amount of carbohydrates consumed we also explored correlations between the different dietary assessment methods for carbohydrate intakes, with results in the range of *r* = 0.57 (fibres) up to *r* = 0.64 (total carbohydrates) when comparing both FFQs and de-attenuated *r* = 0.60 (mono-and disaccharides) up to de-attenuated *r* = 0.75 (polysaccharides) when comparing the GI-FFQ with the 2-day 24 h-recalls. Four of the previously published validation studies on GI/GL also explored correlations and cross-classification of the intake of total carbohydrate and/or carbohydrate-fractions. The Swedish study observed an de-attenuated correlation of *r* = 0.76 for total carbohydrates, where 74% of the population was classified to the same or adjacent quintile [12]. Total carbohydrates, sugar and fibre showed correlations of *r* = 0.55 (39% same quintile), *r* = 0.53 (28% same quintile) and *r* = 0.82 (45% same quintile) in the BMES study [9]. A correlation of *r* = 0.66 (33% same quintile) (women) and 0.72 (51% same quintile) (men) was observed for total carbohydrates in the Japanese study [13]. Finally, total carbohydrates showed correlations of *r* = 0.51 (70% same/adjacent quintile) (men) and *r* = 0.54 (75% same or adjacent quintile) (women) in the Finnish study; correlations for total sugars, fibre and soluble polysaccharides were *r* = 0.27, *r* = 0.67 and *r* = 0.66 in men and *r* = 0.37, *r* = 0.58 and *r* = 0.55 in women, respectively [11].

Comparisons between food groups as assessed with the GI-FFQ versus the 2-day 24 h-recalls in our study resulted in strong de-attenuated correlations for coffee, cake and cookies, soup, bread and dairy. Moderate de-attenuated correlations were observed for fruit, alcoholic beverages, fruit juices, sweets, breakfast cereals and potatoes. The Swedish study was the only other study exploring correlations for food groups comparable to our methods (albeit with differently defined food groups) and strong correlations were revealed for oatmeal (*r* = 0.82), sugar (*r* = 0.80) and breakfast cereals (*r* = 0.70) and moderate correlations for white bread (r = 0.68), sweet-bread or coffee cake (*r* = 0.66), apples or pears (*r* = 0.64), low-alcohol beer (*r* = 0.62), pancakes or waffles (*r* = 0.60), crispbread (*r* = 0.57), rice (*r* = 0.55), fruit stew or soup (*r* = 0.55), biscuits or crackers (*r* = 0.54), jam or marmalade (*r* = 0.53), soft drinks (*r* = 0.51) and whole-grain bread (*r* = 0.50) [12]. Overall, based on these comparisons, we conclude that our results for GI and GL, carbohydrates and food groups are rather similar to results of earlier validation studies in other populations.

Nevertheless, several general as well as study-specific methodological issues need to be addressed. First, our analyses were conducted using data of a subsample (*n* = 475 of 2048) of the NDARD database and NQplus study. Compared to the total population, the mean age (55 vs. 51 years) and the percentage of men (55% vs. 52%) in our subsample is somewhat higher. Other characteristics, including BMI, waist circumference, education level and smoking status, do not substantially differ between the total population and our subsample [26]. It should also be noted, that our population is relatively highly educated (66% with a high educational level) and as such it does not represent the general Dutch population (27% with a high educational level) (as discussed by [26,31]). Previous analyses within this study sample showed that attenuation factors were higher among those with a higher educational attainment [44]. Second, in most previous validation studies, the energy-adjusted correlations/cross-classification seem to be the best validity indicators. While the mean intakes for carbohydrates, several carbohydrate-rich food groups and GL in our study did not substantially change following energy-adjustment using the residual method, the correlation coefficients considerably attenuated, owing to the characteristics of GI-FFQ. The GI-FFQ has been specifically designed to assess GI and GL and therefore it mostly includes carbohydrate-rich foods and consequently, fat and protein rich foods are “under-reported.” We therefore consider energy adjustment in our study to be less suitable and focused our discussion on the “crude” correlations and cross-classification results. Third, to validate the GI-FFQ, a validated general-FFQ and 2-day 24 h-recalls were used as reference methods. All three methods rely on memory of the participants and are analysed using the same food composition tables. Thus, related errors are likely to exist, which may overestimate the validity of the questionnaire. Validated biomarkers for GI and GL are currently lacking. Although previous data have indicated that low-GI diets relate to lower HbA1c concentrations in diabetes patients [45], explorative analyses using HbA1c concentrations in our study did not show promising results. This may be explained by the fact that our study population comprised mainly healthy individuals, of which only 3% had diabetes. In non-diabetics, peak glucose responses following food intake are usually followed by dynamic falls below baseline levels. As such, the total effect of foods on average glucose and HbA1c concentrations in this non-diabetic population may be neutral, in contrast to a diabetic population with a higher level of insulin resistance, where the total effect on average glucose and HbA1c concentrations may be more pronounced. Fourth, foods are usually consumed as a mixture. Aside from carbohydrates, GI may also be influenced by fats and proteins that are simultaneously consumed and as such, the utility of GI values for single foods has been debated [46]. However, Wolever and colleagues revealed that the carbohydrate content and GI of mixed meals explained around 90% of the variation in glycaemic response, with fat and protein having a negligible effect [47].

Study specific limitations include the fact that the GI table used in our study was largely based on American and Australian food items and that data on variety differences and ripeness of plant-based foods was limited [14]. Moreover, the time frame between the three dietary assessment methods was rather large; the general-FFQ was administered at baseline, the GI-FFQ was administered during the summer of 2015 and the two recalls were randomly drawn during a period of 2 years. As the correlation does not necessarily provide information on the ability of an FFQ to adequately rank individuals according to their dietary intake, a strength of this study is that we used cross-classification analyses, correlations, as well as Bland and Altman plots to investigate the validity of the GI-FFQ [48]. Other strengths include the relatively large sample size and the fact that we examined the validity of GI and GL-related factors such as carbohydrate-fractions and a variety of food groups.

To conclude, the validation results of the GI-FFQ seem to be comparable to the results of other studies in this research area as well as to other nutritional factors commonly studied using FFQ, which supports the use of the GI-FFQ to estimate dietary GI and GL and related dietary factors.

## Figures and Tables

**Table 1:** General characteristics of the study population included in the glycaemic index FFQ (GI-FFQ) validation study ^1^.

	All (*n* = 475)	Men (*n* = 263)	Women (*n* = 212)
Age, years	55 ± 11	57 ± 10	53 ± 11
Men, *n* (%)	263 (55)	263 (100)	0 (0)
BMI, kg/m^2^	25.5 ± 3.7	25.9 ± 3.4	25.2 ± 4.0
BMI-category			
<25 kg/m^2^	231 (49)	114 (43)	117 (55)
≥25 kg/m^2^	244 (51)	149 (57)	95 (45)
Waist circumference, cm	90.7 ± 11.9	95.1 ± 10.4	85.2 ± 11.3
Education, *n* (%)			
Low	28 (6)	16 (6)	12 (6)
Medium	132 (28)	65 (25)	67 (31)
High	314 (66)	182 (69)	132 (63)
Smoking status, *n* (%)			
Never	231 (52)	114 (46)	117 (60)
Former	179 (40)	111 (45)	68 (35)
Current	35 (8)	24 (9)	11 (5)
Diabetes, *n* (%)	12 (3)	8 (3)	4 (2)
Diet during past month, *n* (%)	31 (7)	14 (5)	17 (8)

^1^ Missing values: smoking (*n* = 30), education (*n* = 1), diet (*n* = 1). BMI: body mass index.

**Table 2:** Absolute intakes of nutrients, foods, glycaemic index (GI) and glycaemic load (GL) as measured by the general-FFQ, glycaemic index FFQ (GI-FFQ) and 2-day 24 h-recalls.

		GI-FFQ		General-FFQ		2-day 24 h-Recall	
	GI ^1^	Mean	SD	Mean	SD	Mean	SD
Energy, kJ/day	-	5854	1725	9054	2113	9084	2028
Carbohydrates, g/day	-	214	65	234	63	237	64
Mono/disaccharides, g/day	-	100	39	103	33	107	38
Polysaccharides, g/day	-	114	36	132	41	130	39
Fibres, g/day	-	20	6	25	7	24	7
Bread, g/day	63	127	60	137	58	149	69
Breakfast cereals, g/day	54	8	14	14	35	11	19
Potatoes, g/day	85	62	39	82	57	103	98
Pasta, g/day	46	27	22	26	24	49	99
Rice, g/day	72	24	22	31	29	36	79
Vegetables, g/day	20	110	71	156	83	166	104
Fruit, g/day	47	207	126	193	126	203	136
Dairy, g/day	33	331	210	352	182	354	186
Soup, g/day	42	44	43	44	53	98	150
Coffee, g/day	62	486	287	466	312	465	293
SSB, g/day	62	52	115	25	61	89	178
ASB, g/day	11	25	97	22	67	43	134
Fruit juices, g/day	54	89	121	61	75	106	160
Alcoholic beverages, g/day	89	75	145	154	196	194	247
Savoury snacks, g/day	57	44	41	39	37	22	41
Cake/cookies, g/day	61	38	28	36	27	52	43
Sweets, g/day	52	33	28	32	24	33	25
GI	-	54	3	53	4	53	5
GL	-	117	37	126	38	127	37

^1^ Mean GI of the included food items in the GI-FFQ; SSB: sugar sweetened beverages; ASB: artificially sweetened beverages.

**Table 3:** Validation results of the glycaemic index FFQ (GI-FFQ) with the general-FFQ and 2-day 24 h-recalls.

	GI-FFQ with General-FFQ			GI-FFQ with 2-day 24 h-Recalls					
	Similar Q or T	Crude	Energy-Adjusted	Similar Q or T	Crude	ICC	De-Attenuated	Energy-Adjusted	Energy Adjusted De-Attenuated
	%	*r*	*r*	%	*r*		*r*	*r*	*r*
Energy, kJ/day	46	0.58	-	39	0.43	0.52	0.60	-	
Carbohydrates, g/day	48	0.64	0.34	40	0.53	0.63	0.67	0.27	0.34
Mono/disaccharides, g/day	45	0.60	0.45	41	0.48	0.63	0.60	0.41	0.52
Polysaccharides, g/day	48	0.61	0.31	44	0.57	0.58	0.75	0.35	0.46
Fibres, g/day	44	0.57	0.60	41	0.52	0.67	0.64	0.48	0.59
Bread, g/day	54	0.71	0.61	46	0.61	0.67	0.75	0.49	0.60
Breakfast cereals, g/day	62	0.41	0.46	62	0.43	0.70	0.51	0.42	0.50
Potatoes, g/day	43	0.55	0.49	33	0.27	0.29	0.50	0.25	0.46
Pasta, g/day	43	0.56	0.51	-	0.15	0.10	0.47	0.11	0.35
Rice, g/day	45	0.45	0.45	-	0.14	0.09	0.47	0.16	0.53
Vegetables, g/day	41	0.47	0.47	32	0.20	0.38	0.32	0.19	0.31
Fruit, g/day	52	0.67	0.67	40	0.48	0.65	0.60	0.49	0.61
Dairy, g/day	45	0.58	0.58	45	0.58	0.66	0.71	0.56	0.69
Soup, g/day	37	0.46	0.45	48	0.24	0.10	0.76	0.20	0.63
Coffee, g/day	49	0.79	0.76	55	0.76	0.82	0.84	0.75	0.83
SSB, g/day	58	0.50	0.41	51	0.32	0.48	0.46	0.31	0.45
ASB, g/day	-	0.44	0.51	-	0.28	0.66	0.34	0.33	0.41
Fruit juices, g/day	51	0.59	0.56	46	0.40	0.51	0.56	0.33	0.46
Alcoholic beverages, g/day	53	0.67	0.65	42	0.47	0.64	0.59	0.41	0.51
Savoury snacks, g/day	39	0.44	0.37	40	0.22	0.12	0.64	0.16	0.46
Cake/cookies, g/day	47	0.62	0.55	36	0.42	0.30	0.77	0.41	0.75
Sweets, g/day	42	0.45	0.44	37	0.37	0.48	0.53	0.44	0.64
GI	43	0.58	0.53	35	0.40	0.39	0.64	0.38	0.61
GL	48	0.65	0.30	44	0.57	0.59	0.74	0.27	0.35

ICC: Intra class Correlation Coefficient; SSB: sugar sweetened beverages; ASB: artificially sweetened beverages. ASB intake data as obtained by the GI-FFQ did not allow cross-classification analyses. Pasta and rice intake data as obtained by the 2-day 24 h-recalls did now allow cross-classification analyses. Limited variation in intake data of breakfast cereals as obtained by the GI-FFQ resulted in analyses per tertiles instead of quartiles. Limited variation in intake data of SSBs as obtained by the general-FFQ and 2-day 24 h-recalls resulted in analyses in tertiles instead of quartiles. Limited variation in intake data of fruit juices, savoury snacks and soups as obtained by 2-day 24 h-recalls resulted in analyses in tertiles instead of quartiles.

**Table 4:** Validation results of glycated haemoglobin (HbA1c) blood concentration with reported intakes of GI, GL in the 24 h- recalls.

	GI-FFQ with HbA1c				General-FFQ with HbA1c				24 h-Recalls with HbA1c					
	Crude Spearman		Same Quartile	Adjacent Quartile	Crude Spearman r		Same Quartile	Adjacent Quartile	Crude Spearman r		De-Attenuated		Same Quartile	Adjacent Quartile
	*r*	*P*	%	%	*r*	*P*	%	%	*r*	*P*	ICC	*r*	%	%
Carbohydrates	0.00	0.97	26	36	−0.03	0.56	28	35	−0.04	0.40	0.63	−0.05	23	38
Mono- and disaccharides	0.02	0.62	24	41	0.00	0.99	22	41	−0.05	0.28	0.63	−0.06	23	38
Polysaccharides	−0.02	0.72	26	36	−0.05	0.26	28	34	0.00	0.94	0.58	−0.01	25	37
Fibres	−0.02	0.70	27	35	0.02	0.72	27	39	−0.02	0.72	0.67	−0.02	25	35
GI	−0.09	0.04	23	36	−0.04	0.34	25	36	0.04	0.35	0.39	0.07	26	40
GL	−0.02	0.74	28	34	−0.03	0.49	26	37	−0.02	0.61	0.59	−0.03	25	36

## Supplementary Materials

The following are available online at http://www.mdpi.com/2072-6643/11/1/13/s1, Table S1: List of food items identified as key factors to assess dietary GI/GL, Table S2: Validation results for the GI-FFQ vs. general-FFQ stratified for sex.
