# Supplementary material for: The Glycaemic Index-Food-Frequency Questionnaire: Development and Validation of a Food Frequency Questionnaire Designed to Estimate the Dietary Intake of Glycaemic Index and Glycaemic Load: An Effort by the PREVIEW Consortium
Source: Nutrients. 2018 Dec 20;11(1):13. doi: 10.3390/nu11010013 (PMC6356369; doi:10.3390/nu11010013)
Supplement: Supplementary file 1 [file nutrients-11-00013-s001.pdf]

Table S1. List of food items identified as key factors to assess dietary GI/GL.

Food group	Food item
**Potatoes**	Potatoes fried
	Potatoes sliced frozen unprepared
	Potatoes without skins boiled
	French fries
**Beverages**	
Alcoholic beverages	Beer
	Wine (red, white, rose)
Sugar-sweetened beverages	Soft drink with and without caffeine
	Ice tea
	Lemonade squash Dubbelfriss light
	Fruit drink concentrate (undiluted, diluted)
	Whey drink Taksi with sugar
	Juice drink (Dubbelfriss, Vruchtenfris, Tintelfruit, light)
Juices	Orange juice (fresh, pasteurized)
	Apple juice
	Fruit juice with >2 fruits
**Bread**	
Rusks, crackers	Crispbakes (plain, wholemeal)
	Crispbread wholemeal
	Toast
Breads	Bread brown wheat
	Bread wholemeal (with or without seeds)
	Bread multigrain (with or without seeds)
	Bread white (water- , milk-based)
	Roll white (soft, hard)
	Roll brown (soft, hard)
	Bun currant/raisin
	Croissant
	Pita
	Baguette white
	Bread crumbs
**Fruit**	Apple (with and without skin)
	Banana
	Orange
	Grapes
	Melon
	Kiwi fruit
	Apple sauce tinned
**Cakes and cookies**	Cake spiced Dutch (ontbijtkoek)
	Cake sponge Dutch (eierkoek)
	Cupcake iced
	Apple pie Dutch
	Biscuit (spiced, wholemeal, plain)
	Biscuit filled (with chocolate, fruit)
	Children’s biscuit average
	Chocolate chip cookie
**Cereals**	Muesli with fruit
	Muesli crunchy (plain, with fruit)

	Pasta plain boiled
	Rice boiled (white, brown)
**Vegetables**	Zucchini
	Cucumber
	Carrots
	Onion
	Pea garden
	Lettuce iceberg
	Sweet corn
	French beans
	Sweet pepper green
**Sandwich filling**	Peanut butter (with or without nut pieces)
	Chocolate spread (duo, hazelnut)
	Chocolate confetti plain
	Coloured confetti fruit-flavoured
	Jam
**Dairy (excluding cheese)**	Milk (skimmed, semi-skimmed, whole)
	Buttermilk
	Chocolate milk
	Yogurt (low, half, full fat)
	Yogurt drink (with or without sweeteners)
	Custard (all flavours, full-fat)
	Whipped cream (with or without sugar)
	Ice cream dairy-based
**Nuts, seeds, snacks**	Nuts and seeds
	Mixed nuts, raisins, and currants
	Crisps potato (flavoured, unflavoured)
	Dough for pizza and savoury pie
	Pancake
**Sugar and confectionary**	Boiled sweets
	Wine gums
	Liquorice (sweet, salted)
	Sugar granulated
	Waffle syrup
	Ice lolly / sorbet
	Candybar
	Chocolate (white, milk, dark)

Note that for the final FFQ, food items were grouped when there was not a large variation between their GI. For example, vegetables largely have the same GI; therefore, we did not assess all types of vegetables separately.

Supplemental Table 2. Validation results for the GI-FFQ vs. general-FFQ stratified for sex.

	Men (n=263)				Women (n=212)			
	Absolute intakes		similar Q or T	Crude	Absolute intakes		similar Q or T	Crude
	mean	SD	%	*r*	Mean	SD	%	*r*
Energy, kJ/day	6315	1730	46	0.51	5281	1539	46	0.56
Carbohydrates, g/d	231	64	48	0.60	193	59	47	0.62
Mono/disaccharides, g/d	106	40	45	0.60	92	37	45	0.55
Polysaccharides, g/d	125	36	45	0.57	101	31	52	0.59
Fibres, g/d	21	6	43	0.56	19	5	45	0.58
Bread, g/d	143	62	53	0.71	108	52	56	0.67
Breakfast cereals, g/d	7.5	14	64	0.45	8	13	58	0.36
Potatoes, g/d	72	43	43	0.55	50	29	43	0.48
Pasta, g/d	29	23	43	0.60	24	19	44	0.51
Rice, g/d	26	25	45	0.43	22	18	45	0.47
Vegetables, g/d	98	73	37	0.39	126	66	47	0.52
Fruit, g/d	204	126	51	0.66	210	127	54	0.67
Dairy, g/d	345	225	47	0.63	314	189	43	0.50
Soup, g/d	42	43	36	0.45	46	43	37	0.46
Coffee, g/d	558	293	50	0.72	396	254	47	0.82
SSB, g/d	66	128	59	0.52	35	94	56	0.41
ASB, g/d	18	68	-	0.44	32	123	-	0.44
Fruit juices, g/d	99	119	52	0.64	77	124	50	0.52
Alcoholic beverages, g/d	116	167	56	0.73	25	90	48	0.41
Savoury snacks, g/d	48	44	41	0.49	38	35	35	0.36
Cake/cookies, g/d	38	29	44	0.61	38	27	51	0.63
Sweets, g/d	35	27	43	0.57	30	28	41	0.55
GI	55	3	47	0.51	53	3	48	0.55
GL	128	37	45	0.58	103	33	41	0.62

SSB: sugar sweetened beverages; ASB: artificially sweetened beverages; ASB intake data as obtained by the GI-FFQ did not allow cross-classification analyses.
